# Integral membrane protein 2A enhances sensitivity to chemotherapy via notch signaling pathway in cervical cancer

**DOI:** 10.1080/21655979.2021.2001218

**Published:** 2021-12-07

**Authors:** Yan Li, Jianhua Wang, Chengzhen Gao, Qiyan Hu, Xiaogang Mao

**Affiliations:** a Department of Obstetrics and Gynecology, The Yancheng Clinical College of Xuzhou Medical University, the First People’s Hospital of Yancheng, Yancheng, China; b Department of Gastroenterology, The Yancheng Clinical College of Xuzhou Medical University, the First People’s Hospital of Yancheng , Yancheng, China; c Department of Oncology, Xiangyang Central Hospital, Affiliated Hospital of Hubei University of Arts and Science, Xiangyang City, China; d Department of Obstetrics and Gynecology, Xiangyang Central Hospital, Affiliated Hospital of Hubei University of Arts and Science, Xiangyang City, China

**Keywords:** Cervical cancer, ITM2A, cisplatin, chemotherapy, resistance, Notch signaling pathway

## Abstract

As the second most common cancer among women, cervical cancer is a huge threat to their health all over the world. Integral membrane protein 2A (ITM2A), a member of the Type II Integral Membrane protein (ITM2) family, has been reported to act as a tumor suppressor in breast cancer and ovarian cancer. Moreover, the low expression of ITM2A was associated with cervical adenocarcinoma. However, the function of ITM2A in drug resistance in cervical cancer remains unclear. Here, we used bioinformatics methods to screen differentially expressed genes (DEGs) closely related to chemotherapeutic relapse cervical carcinoma. ITM2A is downregulated in cervical tumor tissues and is associated with poor survival. Furthermore, ITM2A is also downregulated in cervical cancer cells with cisplatin resistance. Overexpression of ITM2A increases the cisplatin sensitivity of cervical cancer cells. Mechanically, ITM2A upregulation mediates the sensitivity of cervical cancer cell through Notch signaling pathway. Our study suggests that ITM2A may serve as a target in mediating cisplatin-resistant cervical cancer.

## Introduction

Cervical cancer is the second most common cancer among women and the most common cause of death from gynecological cancer worldwide [1,2–4]. At the same time, the survival rate in patients with advanced or recurrent cervical cancer is still poor, with a 1-year survival rate of 10–20% [5]. Up to now, chemotherapy is still the first choice for the treatment of cervical cancer. The drug resistance of tumor cells is the main reason for chemotherapy failure, tumor recurrence, and even patient death [6–8]. Therefore, it is urgent to study the mechanism of drug resistance and prevent or reverse drug resistance.

Integral membrane protein 2A (ITM2A) consists of 263 amino acid residues with evolutional conservation between human and mouse. ITM2A, along with ITM2B and ITM2C, belongs to the Type II Integral Membrane protein family. This family contains a BRICHOS domain with the activity of a chaperone. Previous study reported that ITM2A is associated with cell differentiation and is cell-type dependent. In addition, Tai et al. demonstrated that ITM2A is a target of GATA-3 and is transactivated by GATA-3, which plays an important role in regulating the function of T cells [9]. Sim and colleagues also demonstrated that ITM2A is a transcriptional target of PKA-CREB, and the expression of ITM2A is regulated by PKA-CREB signaling pathway, which is involved in autophagic flux by interacting with v-ATPase [10]. A recent study revealed that ITM2A acts as a tumor suppressor in many epithelial cancers, including breast cancer, ovarian cancer, and cervical cancer. However, the function and mechanism of ITM2A in drug resistance in cervical cancer has not been completely clarified.

More and more evidence show that the imbalance of ITM2A expression is closely related to tumors [11]. Therefore, ITM2A may also exert an important role in cervical cancer development and treatment. This study aims to detect the expression of ITM2A in cervical cancer tissues and cells with cisplatin treatment, and the underlying mechanism of ITM2A in cisplatin resistance of cervical carcinoma. The results showed that the expression of ITM2A was significantly downregulated in cervical cancer tissue specimens and cells resistant to cisplatin chemotherapy. Overexpression of ITM2A increases the sensitivity of cervical cancer to cisplatin. High expression of ITM2A mediated by cisplatin drug stress can promote the death of CESC cells and make it more sensitive to the cytotoxic effects of cisplatin. These findings indicate that ITM2A may be a potential target to decrease recurrence after CESC chemotherapy.

## Materials and methods

### Cells, reagents, and tissues

Hela and SIHA were cultured in DMEM (purchased from Procell Company, Wuhan, China) supplemented with 10% FBS and 100 U/ml penicillin/streptomycin. siITM2A and cisplatin were purchased from GeneCopoeia. Cervical cancer specimens (40 cases) were provided by The First People’s Hospital of Yancheng and approved by the Ethics Committee of the First People’s Hospital of Yancheng. The experimental manipulations performed have been previously reported [12]. The siRNA of ITM2A was shown as follows: *siRNA1#:GAGCCTGCATTTACAAGTACTTCAT; siRNA2#:TGGCAGATATCTGCCTCAAACTTAT*. The gene ITM2A was cloned into pcDNA3.1 (+) expression vector (Invitrogen, USA). Lipofectamine 3000 was used to co-transfect plasmids for the construction of ITM2A overexpression cells.

### Cell viability and colony formation assays

Cell proliferation viability and cytotoxicity assay were performed as described previously [13]. Cells were seeded, and then the colorimetric MTT Assay kit (Roche Diagnostics) was used to measure the cell proliferation until 72 hours. For the foci formation assay, cells were seeded, cultured, fixed, and stained.

### Immunohistochemical assay

The immunohistochemical assay was performed as previously described [14]. Tumor sections were deparaffinized, blocked, incubated, counterstained, and photographed. The images were captured using a microscope.

### RNA extraction and real-time quantitation PCR

Total RNA was extracted as previously depicted [15]. Primers were listed as follows:

ITM2A forward: 5ʹ-AATGACTGCTTACCTGGACTTG-3ʹ;

ITM2A reverse: 5ʹ-TCCACAGCAACTAGGTCTTCT-3ʹ;

NOTCH1 forward: 5ʹ-TGGACCAGATTGGGGAGTTC-3ʹ;

NOTCH1 reverse: 5ʹ-GCACACTCGTCTGTGTTGAC-3ʹ;

Hes1 forward: 5ʹ-CCTGTCATCCCCGTCTACAC-3ʹ;

Hes1 reverse: 5ʹ-CACATGGAGTCCGCCGTAA-3ʹ;

Jagged1 forward: 5ʹ-TGTGGCTTGGATCTGTTGCTTGG-3ʹ;

Jagged1 reverse: 5ʹ-ACGTTGTTGGTGGTGTTGTCCTC-3ʹ;

c-Myc forward: 5ʹ-GTCAAGAGGCGAACACACAAC-3ʹ;

c-Myc reverse: 5ʹ-TTGGACGGACAGGATGTATGC-3ʹ;

GAPDH forward: 5ʹ- AGACAGCCGCATCTTCTTGT-3ʹ;

GAPDH reverse: 5ʹ- CTTGCCGTGGGTAGAGTCAT-3ʹ.

### Western blotting

Western blotting was performed as previously depicted [15]. The membranes were immunoblotted with the following antibodies: anti-mouse ITM2A (1:1000, Abcam, ab279387, England), anti-mouse P-gp (1:1000, Santa, sc-365930, USA), anti-mouse MDR-1 (1:1000, Santa, sc-55510, USA), anti-mouse BCRP (1:1000, Santa, sc-377176, USA), anti-rabbit c-NOTCH1 (1:1000, sigma, SAB4502019, Germany), anti-mouse Hes1 (1:1000, Santa, sc- 166410, USA), anti-mouse Jagged1 (1:1000, Santa, sc- 390177, USA), anti-mouse c-Myc (1:1000, Santa, sc-40, USA), and anti-mouse β-actin (1:1000, Santa, sc-47724, USA).

### Cisplatin-resistant cell lines construct

The construction of cisplatin-resistant cells was performed as previously depicted [16], adopting to high-dose shock combined with a concentration gradient method. First, the median lethal concentration (IC50) of cisplatin was determined in cervical cancer cells Hela and SIHA. The cells were treated with 1:1000 cisplatin as the initial concentration of IC50 for 24 h, washed with normal saline for 3 times, and routinely withdrew the drug cultivated. The medium was changed every day to remove dead cells. After about 7–18 days, the growth of live cells will resume. After entering the logarithmic growth phase, the cells passaged once, and continued to culture until the growth was good and stable. The concentration of cisplatin will be gradually induced (gradient 10 ng/ml).

### Volcano plot, Kaplan–Meier, and GO-KEGG analysis

Different expression genes from GEO were determined by bioinformatics analysis (limma analysis of different expression genes, data filtering, and standardization were performed as described previously [17]). After preprocessing the cervical cancer data from TCGA, we extracted TPM-type tumor expression data. According to the expression level of ITM2A, high and low groups (median) were divided. The results of differentially expressed gene analysis were performed by limma and performed by KEGG-GO analysis by using of the clusterProfiler package for R language [18,19]. Pathway enrichment analysis was derived from metascape online (https://metascape.org/gp/index.html) [20]. The Kaplan–Meier curve tool (http://kmplot.com/analysis/) was used to measure the survival rate of patients.

### Statistical analysis

Statistical analyses were performed using one-way ANOVA. Data were presented as means ± SEM of at least three independent experiments. A P-value of 0.05 or less was considered to be statistically significant.

## Results

ITM2A, as a member of the Type II Integral Membrane protein (ITM2) family, has been reported to exert a tumor suppressor role in breast cancer and ovarian cancer. Moreover, the low expression of ITM2A was associated with cervical adenocarcinoma. We speculated that ITM2A may also play a critical role in drug resistance of cervical cancer. We chose the key gene-ITM2A of DEGs from TCGA by bioinformatics analysis, and then we analyzed the expression of ITM2A in cervical cancer cells and tissues. The relationship between ITM2A and survival in chemotherapeutic relapse cervical carcinoma was also analyzed. The expression of drug-resistance-related mRNA and protein was determined by RT-qPCR and western blotting, respectively. Cell viability and cell growth were measured by MTT assay and clonogenic assay. Our results indicated that ITM2A negatively regulates cisplatin resistance in cervical cancer through Notch signal pathway.

### Screening of DEGs associated with chemotherapeutic relapse

**Figure 1.**
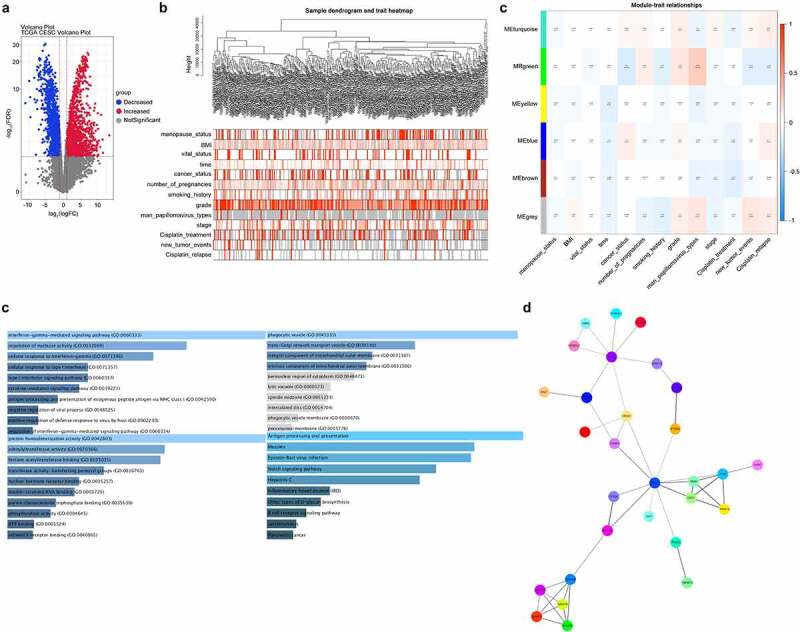
Screening of DEGs associated with chemotherapeutic relapse from TCGA. (a) Venn plots analyzed the DEGs in cervical cancer from TCGA. Red: increased DEGs, blue: decreased DEGs. (b) WGCNA cluster analysis of DEGs significantly associated with chemotherapeutic relapse. (c) Function and pathway enrichment analysis of ITM2A in the green-module gene set in WGCNA. (d) Protein–protein interaction network of differential genes was determined by STRING

First, we analyzed the different expression genes by limma&edgeR from TCGA_CESC data. As shown in Figure 1(a), 865 increased DEGs (red) and 1177 decreased DEGs (blue) were analyzed in volcano plot. We further analyzed chemotherapeutic relapse-related genes by WGCNA cluster analysis, and screened 45 DEGs that were significantly associated with chemotherapeutic relapse (Figure 1(b)). ITM2A was a decreased DEGs in green module and associated with notch signaling pathway (Figure 1(c,d)).

### The expression of ITM2A was significantly downregulated in patients with recurrent cervical cancer after clinical cisplatin treatment

**Figure 2.**
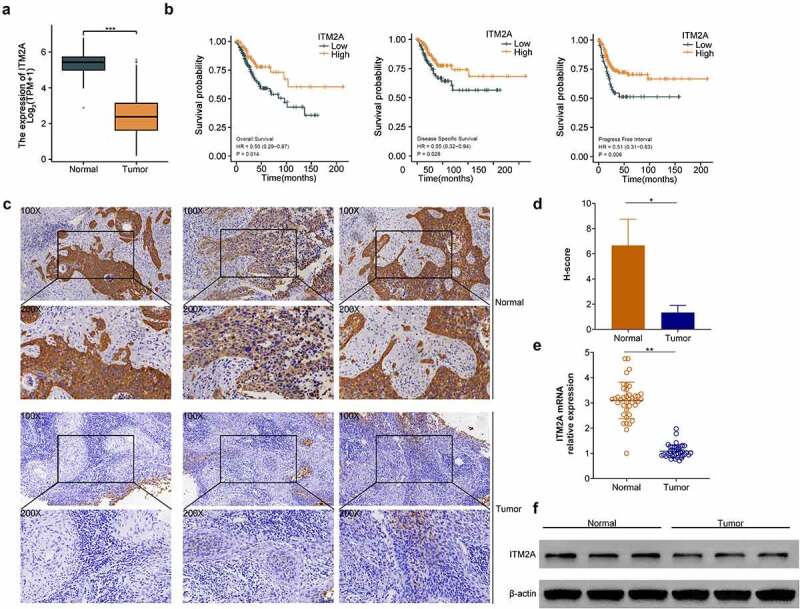
The expression of ITM2A was significantly downregulated in patients with recurrent cervical cancer after clinical cisplatin treatment. (a) mRNA expression of ITM2A was analyzed in TCGA. ***P < 0.001. (b) Kaplan–Meier curve analyzed the relationship between ITN2A expression and survival rate (including OS: overall survival, DSS: disease-specific survival, PFI: progress-free interval) in recurrence cases after treatment with cisplatin. (c) Representative images of immunohistochemical staining of ITM2A in normal and tumor tissues. (d&e) The expression of ITM2A in normal and tumor tissues was determined by RT-qPCR (d) and western blotting (e). Error bars represent data from three independent experiments (mean ± SD). **P < 0.01

Next, we analyzed the mRNA expression of ITM2A in cervical cancer from TCGA database. As shown in Figure 2(a), ITM2A was significantly downregulated in tumor tissues compared with adjacent normal tissues. Moreover, compared with the low ITM2A group, patients with high expression of ITM2A showed better survival rate, including OS, DSS, and PFI (Figure 2(b)). Then, we collected 40 cases of cervical cancer relapse specimen from patients treated with cisplatin and measured ITM2A protein using IHC staining. The expression of ITM2A was decreased in tumor tissues of cervical cancer with cisplatin treatment compared with normal tissues (Figure 2(c)). We also analyzed the mRNA and protein expression of ITM2A using RT-qPCR (Figure 2(d)) and western blotting (Figure 2(e)). ITM2A was downregulated in tumor compared with normal specimens. Collectively, these data indicated that the expression of ITM2A is downregulated in cervical cancer after cisplatin treatment.

### The expression of ITM2A was significantly downregulated in cisplatin-resistant cervical cancer cells

**Figure 3.**
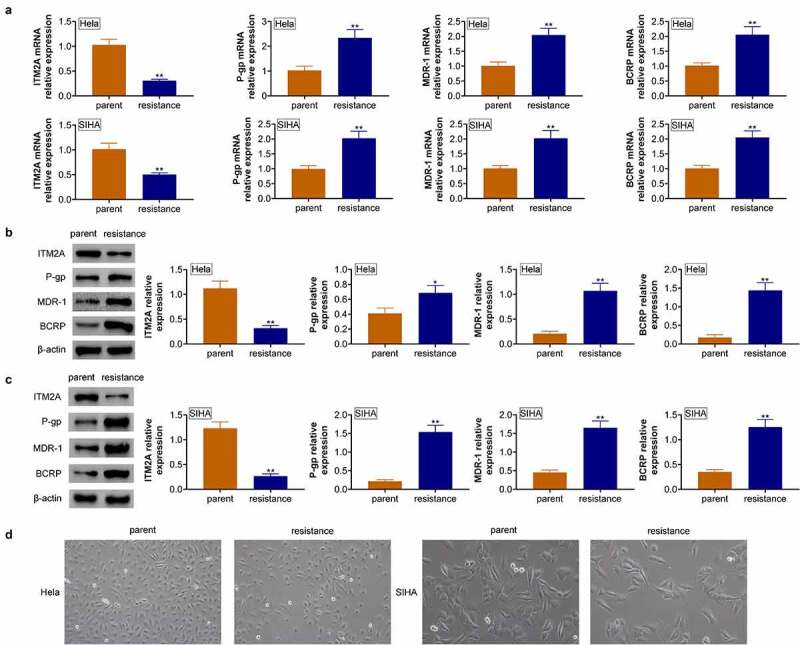
The expression of ITM2A was significantly downregulated in cisplatin-resistant cervical cancer cells. (a) RT-qPCR was used to measure the expression of drug-resistance-related genes in naive cells and cisplatin cells of Hela and SIHA. (b&c) The expression of drug-resistance-related proteins in naive cells and cisplatin cells of Hela (b) and SIHA (c) was determined by western blotting. (d) Morphology of parent cells and PTX resistance cells in MDA-MB231 and MDA-MB436 was measured by microscopy. Error bars represent data from three independent experiments (mean ± SD). *P < 0.05, **P < 0.01

To clarify the function of ITM2A in cervical cancer, we also analyzed the expression of ITM2A in cisplatin-resistant cervical cancer cell lines. As shown in Figure 3(a), ITM2A mRNA was downregulated in cisplatin resistance cells of Hela and SIHA. Furthermore, the mRNA expressions of P-gp, MDR-1, and BCRP were significantly upregulated in Hela/cisplatin compared with normal Hela, and similar results were shown in SIHA cell lines. We also analyzed drug-resistance-related protein expression. As shown in Figure 3(b,c), P-gp, MDR-1, and BCRP protein expressions were upregulated in cisplatin-resistant cell lines. Furthermore, we found that disintegration and death of cisplatin-resistant Hela and SIHA cells were reduced, and surviving cells gradually returned to their original form (Figure 3(d)). Collectively, our data demonstrated that ITM2A negatively regulates drug-resistance-related protein expression.

### Overexpression of ITM2A increases the cisplatin sensitivity of cervical cancer cells

**Figure 4.**
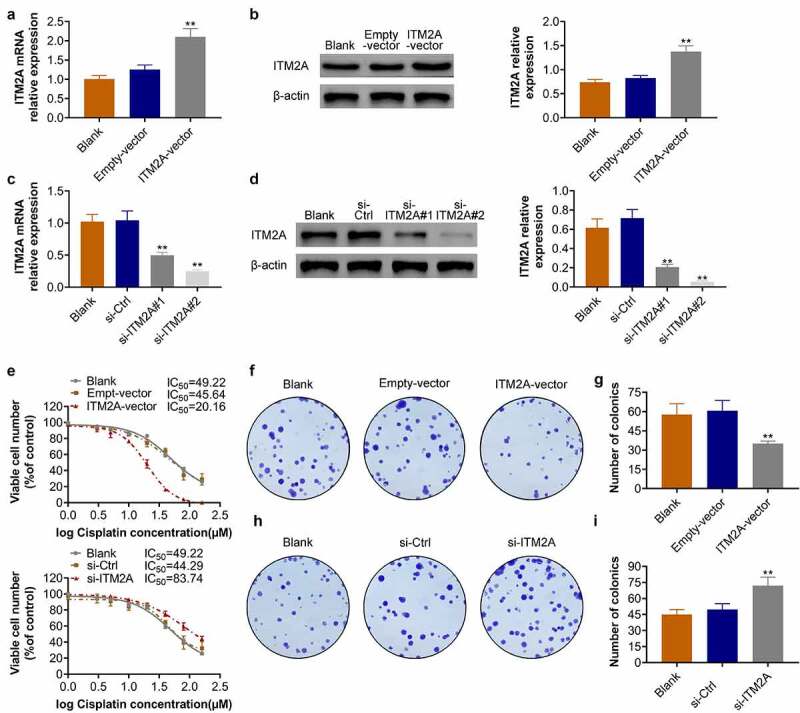
Overexpression of ITM2A increases the cisplatin sensitivity of cervical cancer cells. (A&B) Overexpression of ITM2A in cisplatin-resistant SIHA cells was confirmed by RT-qPCR (a) and western blotting (b). (C&D) Knockdown of ITM2A in cisplatin-resistant SIHA cells was confirmed by RT-qPCR (c) and western blotting (d). (e) SIHA cisplatin-resistant cell lines were transfected with indicated plasmids, after treated with different concentration of cisplatin for 72 h, cell viability was measured by MTT assay. (F&G) Clonogenic assay was performed to measure the capacity of foci formation in SIHA cisplatin-resistant cells treated with empty-vector and ITM2A-vector. (H&I) Clonogenic assay was performed to measure the capacity of foci formation in SIHA cisplatin-resistant cells treated with si-ITM2A and si-ctrl. Results presented represent the means of triplicate experiments ± SEM. **P < 0.01

Next, we constructed ITM2A-overexpression and ITM2A-knockdown cell lines of cisplatin-resistant SIHA. The overexpression of ITM2A in cisplatin-resistant SIHA was confirmed by RT-qPCR (Figure 4(a)) and western blotting (Figure 4(b)). Similarly, the knockdown efficiency of siITM2A (#1 and #2) in SIHA/cisplatin cell lines was also determined by RT-qPCR (Figure 4(c)) and western blotting (Figure 4(d)). We then confirmed the IC50 in ITM2A-overexpression and ITM2A-knockdown SIHA cells. As shown in Figure 4(e), the IC50 of ITM2A-overexpression SIHA cells was 20.16 μM. The IC50 of ITM2A-knockdown SIHA cells was 83.74 μM. In addition, the overexpression of ITM2A decreased colony formation in SIHA/cisplatin cells (Figure 4(f,g)), while knockdown of ITM2A increased colony formation in SIHA/cisplatin cells. (Figure 4(h,i)). Taken together, these data indicated that ITM2A positively regulates the sensitivity of SIHA to cisplatin.

### ITM2A regulates chemotherapeutic drug sensitivity through the notch pathway

**Figure 5.**
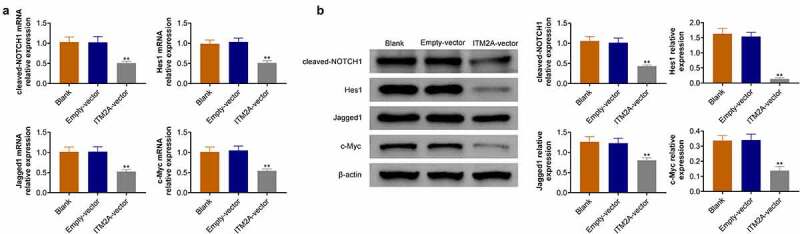
ITM2A regulates chemotherapeutic drug sensitivity through the Notch pathway. (a) The mRNA expression of Notch signaling pathway related genes in ITM2A overexpression cells were measured by RT-qPCR. (b) Western blotting was used to analysis the expression of these protein in ITM2A overexpression cells. Results presented represent the means of triplicate experiments ± SEM. **P < 0.01

The Notch protein family consists of four transmembrane receptors (Notch 1, 2, 3, and 4), which was separated after ligand binding on the cell surface, resulting in activation of downstream signal. Here, we found that ITM2A was associated with a notch signal pathway by bioinformatics analysis. Therefore, we measured the expression of c-Notch1, Hes1, Jagged1, and c-Myc using RT-qPCR and western blotting. As shown in Figure 5(a,b), overexpression of ITM2A reduced the expression of c-Notch1, Hes1, Jagged1, and c-Myc in SIHA cell lines. Taken together, our data indicated that ITM2A regulates chemotherapeutic drug sensitivity through downregulation of Notch signaling.

## Discussion

Cervical cancer is the fourth most common cancer in females with high mortality and morbidity among women worldwide [21]. Cervical cancer is mainly associated with high-risk human Papilloma viruses (PVs), smoking, and diet. Most cervical cancer patients have received chemotherapy, and only a few women achieve good results because of drug resistance, especially, in invasive cervical cancer (ICC) [22]. It is necessary to clarify the molecular mechanism of drug resistance during therapy. In this study, we demonstrated that ITM2A is a decreased DEGs in cervical cancer patients with chemotherapeutic relapse. In addition, low expression of ITM2A is associated with poor survival in cervical cancer patients. The expression of ITM2A is reduced in cervical cancer cells with cisplatin resistance. Moreover, ITM2A negatively regulates the cisplatin sensitivity of cervical cancer cells and inhibits colony formation in cervical cancer cells.

Chemotherapy has been widely used for advanced cervical cancer, combined with radiotherapy and surgical excision for clinical cervical cancer treatment. Although cervical cancer patients have received good treatment with the development of technology and advanced drugs, the prognosis of some patients is still poor because of drug resistance. As is well known, drug resistance is a common spreading phenomenon in cervical cancer treatment. Cisplatin is an important treatment tool against several solid tumors, including (but not limited to) head and neck, ovarian cancer, cervical cancer, and lung cancer [23–26]. Cervical cancer cells are initially susceptible to chemotherapy with cisplatin. Over time, they may be more effective through DNA damage repair, drug inactivation of glutathione and metallothionein, and drug efflux with various transport systems located in the cell membrane. Unfortunately, cancer cells are either inherent or develop resistance to cisplatin relatively quickly, leading to relapse and treatment failure. Therefore, new biomarkers with high specificity and sensitivity for cervical cancer therapy are urgently needed.

Here, we used bioinformatics analysis to screen out ITM2A, a decreased DEGs in cervical cancer with chemotherapy relapse. Previous studies have shown the suppressor role of ITM2A in ovarian cancer and breast cancer [27–29]. In addition, ITM2A deficiency was associated with chemoresistance, including PTX and carboplatin, and ITM2A negatively regulated the IC50 of PTX and carboplatin in ovarian cancer [28]. In this study, we demonstrate that ITM2A positively regulated the sensitivity of cisplatin in cervical cancer cells. Recent study indicated that ITM2A expression was associated with the LC3B-II/LC3B-I ratio in regulating autophagy [10]. Here, we obtain similar results for ITM2A in autophagy regulation in cervical cancer cells.

Mechanically, Notch signaling pathway, a type I transmembrane heterodimeric receptor, induces the transcription of downstream target genes by interacting with its ligands. High levels of Notch signaling pathway molecules have been found in a variety of cancers, leading to enhanced Notch signaling and promoting tumor cell survival. The high expressions of Notch-1 and Jagged-1 are associated with poor prognosis of breast cancer and promote the transformation of breast epithelial cells. Abnormal expressions of Notch-1, Notch-4 or Jagged-1 are common in breast ductal carcinoma and lobular carcinoma. Previous studies demonstrated that the Notch signaling pathway participated in cisplatin resistance by regulating drug detoxification or upregulating DNA repair enzymes. γ-secretase inhibitor inhibited Notch signaling pathway and significantly enhanced the sensitivity of CNE-2 cells to cisplatin [30]. We also found that ITM2A regulates chemotherapeutic drug sensitivity through downregulation of Notch signaling. However, the underlying regulatory mechanism of ITM2A in Notch signaling pathway needs further investigation.

## Conclusion

In summary, we demonstrated that ITM2A is downregulated and associated with poor survival in cervical cancer with chemotherapeutic relapse. Moreover, ITM2A is further downregulated in cisplatin-resistant cervical cancer cells SIHA and Hela. In addition, ITM2A negatively regulates the sensitivity of cisplatin in cervical cancer cells through Notch signaling pathway. However, the molecular mechanisms of ITM2A in regulation of the drug resistance in cervical cancer need further research. ITM2A has the potential to serve as a new marker in the release of drug resistance in the future treatment of cervical cancer.

## Data Availability

All data generated or analyzed during this study are included in this published article.

## Highlight

- Screening of DEGs is associated with chemotherapeutic relapse.
- The expression of ITM2A was significantly downregulated in patients with recurrent cervical cancer after clinical cisplatin treatment.
- The expression of ITM2A was significantly downregulated in cisplatin-resistant cervical cancer cells.
- Overexpression of ITM2A increases the cisplatin sensitivity of cervical cancer cells.
- ITM2A regulates chemotherapeutic drug sensitivity through the Notch pathway.

## References

[1] Zhao M, Huang W, Zou S, et al. A five-genes-based prognostic signature for cervical cancer overall survival prediction. Int J Genomics. 2020;2020:8347639. 32300605 10.1155/2020/8347639 PMC7136791

[2] Sung H, Ferlay J, Siegel RL, et al. Global cancer statistics 2020: GLOBOCAN estimates of incidence and mortality worldwide for 36 cancers in 185 countries. CA Cancer J Clin. 2021;71(3):209–249. 33538338 10.3322/caac.21660

[3] Diaz-Padilla I, Monk BJ, Mackay HJ, et al. Treatment of metastatic cervical cancer: future directions involving targeted agents. Crit Rev Oncol Hematol. 2013;85(3):303–314. 22883215 10.1016/j.critrevonc.2012.07.006

[4] Lorusso D, Petrelli F, Coinu A, et al. A systematic review comparing cisplatin and carboplatin plus paclitaxel-based chemotherapy for recurrent or metastatic cervical cancer. Gynecol Oncol. 2014;133(1):117–123. 24486604 10.1016/j.ygyno.2014.01.042

[5] Waks AG, Winer EP. Breast cancer treatment: a review. Jama. 2019;321(3):288–300. 30667505 10.1001/jama.2018.19323

[6] Shen DW, Pouliot LM, Hall MD, et al. Cisplatin resistance: a cellular self-defense mechanism resulting from multiple epigenetic and genetic changes. Pharmacol Rev. 2012;64(3):706–721. 22659329 10.1124/pr.111.005637 PMC3400836

[7] Lanzi C, Perego P, Supino R, et al. Decreased drug accumulation and increased tolerance to DNA damage in tumor cells with a low level of cisplatin resistance. Biochem Pharmacol. 1998;55(8):1247–1254. 9719480 10.1016/s0006-2952(97)00599-6

[8] Cui Y, Konig J, Buchholz JK, et al. Drug resistance and ATP-dependent conjugate transport mediated by the apical multidrug resistance protein, MRP2, permanently expressed in human and canine cells. Mol Pharmacol. 1999;55(5):929–937. 10220572

[9] Tai TS, Pai SY, Ho IC. Itm2a, a target gene of GATA-3, plays a minimal role in regulating the development and function of T cells. PloS One. 2014;9(5):e96535. 24831988 10.1371/journal.pone.0096535 PMC4022677

[10] Namkoong S, Lee KI, Lee JI, et al. The integral membrane protein ITM2A, a transcriptional target of PKA-CREB, regulates autophagic flux via interaction with the vacuolar ATPase. Autophagy. 2015;11(5):756–768. 25951193 10.1080/15548627.2015.1034412 PMC4509440

[11] Park JS, Jeon EK, Chun SH, et al. ERCC1 (excision repair cross-complementation group 1) expression as a predictor for response of neoadjuvant chemotherapy for FIGO stage 2B uterine cervix cancer. Gynecol Oncol. 2011;120(2):275–279. 21093896 10.1016/j.ygyno.2010.10.034

[12] Yu L, Dong L, Wang Y, et al. Reversible regulation of SATB1 ubiquitination by USP47 and SMURF2 mediates colon cancer cell proliferation and tumor progression. Cancer letters 2019 40–51. 10.1016/j.canlet.2019.01.039 30742943

[13] Dong L, Yu L, Bai C, et al. USP27-mediated Cyclin E stabilization drives cell cycle progression and hepatocellular tumorigenesis. Oncogene. 2018;37(20):2702–2713. 29497124 10.1038/s41388-018-0137-z PMC5955865

[14] Yu L, Dong L, Li H, et al. Ubiquitination-mediated degradation of SIRT1 by SMURF2 suppresses CRC cell proliferation and tumorigenesis. Oncogene. 2020;39(22):4450–4464. 32361710 10.1038/s41388-020-1298-0

[15] Dong L, Yu L, Li H, et al. An NAD(+)-dependent deacetylase SIRT7 promotes HCC development through deacetylation of USP39. iScience. 2020;23(8):101351. 32711345 10.1016/j.isci.2020.101351 PMC7387830

[16] Zhang Y, Shen X. Heat shock protein 27 protects L929 cells from cisplatin-induced apoptosis by enhancing Akt activation and abating suppression of thioredoxin reductase activity. Clin Cancer Res off J Am Assoc Cancer Res. 2007;13(10):2855–2864. 10.1158/1078-0432.CCR-06-2090 17504983

[17] Nagy A, Lanczky A, Menyhart O, et al. Validation of miRNA prognostic power in hepatocellular carcinoma using expression data of independent datasets. Sci Rep. 2018;8(1):9227. 29907753 10.1038/s41598-018-27521-y PMC6003936

[18] Yang YH, Dudoit S, Luu P, et al. Normalization for cDNA microarray data: a robust composite method addressing single and multiple slide systematic variation. Nucleic Acids Res. 2002;30(4):e15. 11842121 10.1093/nar/30.4.e15 PMC100354

[19] Smyth GK, Speed T. Normalization of cDNA microarray data. Methods. 2003;31(4):265–273. 14597310 10.1016/s1046-2023(03)00155-5

[20] Zhou Y, Zhou B, Pache L, et al. Metascape provides a biologist-oriented resource for the analysis of systems-level datasets. Nat Commun. 2019;10(1):1523. 30944313 10.1038/s41467-019-09234-6 PMC6447622

[21] Torre LA, Bray F, Siegel RL, et al. Global cancer statistics, 2012. CA Cancer J Clin. 2015;65(2):87–108. 25651787 10.3322/caac.21262

[22] Li Y, Cui N, Zheng PS, et al. BMX/Etk promotes cell proliferation and tumorigenicity of cervical cancer cells through PI3K/AKT/mTOR and STAT3 pathways. Oncotarget. 2017;8(30):49238–49252. 28514765 10.18632/oncotarget.17493 PMC5564764

[23] Marquard FE, Jucker M. PI3K/AKT/mTOR signaling as a molecular target in head and neck cancer. Biochem Pharmacol. 2020;172:113729. 31785230 10.1016/j.bcp.2019.113729

[24] Zhang HY, Zhang PN, Sun H. Aberration of the PI3K/AKT/mTOR signaling in epithelial ovarian cancer and its implication in cisplatin-based chemotherapy. Eur J Obstet Gynecol Reprod Biol. 2009;146(1):81–86. 19540648 10.1016/j.ejogrb.2009.04.035

[25] Duan W, Liu X. PSAT1 upregulation contributes to cell growth and cisplatin resistance in cervical cancer cells via regulating PI3K/AKT signaling pathway. Ann Clin Lab Sci. 2020;50(4):512–518. 32826249

[26] Chen B, Shen Z, Wu D, et al. Glutathione peroxidase 1 promotes NSCLC resistance to cisplatin via ROS-induced activation of PI3K/AKT pathway. Biomed Res Int. 2019;2019:7640547. 31032363 10.1155/2019/7640547 PMC6457285

[27] Abuderman AA, Harb OA, Gertallah LM. Prognostic and clinicopathological values of tissue expression of MFAP5 and ITM2A in triple-negative breast cancer: an immunohistochemical study. Contemp Oncol. 2020;24(2):87–95. 10.5114/wo.2020.97520 PMC7403766 32774133

[28] Nguyen TM, Shin IW, Lee TJ, et al. Loss of ITM2A, a novel tumor suppressor of ovarian cancer through G2/M cell cycle arrest, is a poor prognostic factor of epithelial ovarian cancer. Gynecol Oncol. 2016;140(3):545–553. 26691219 10.1016/j.ygyno.2015.12.006

[29] Zhang R, Xu T, Xia Y, et al. ITM2A as a tumor suppressor and its correlation with PD-L1 in breast cancer. Front Oncol. 2020;10:581733. 33680917 10.3389/fonc.2020.581733 PMC7928367

[30] Zhou JX, Han JB, Chen SM, et al. gamma-secretase inhibition combined with cisplatin enhances apoptosis of nasopharyngeal carcinoma cells. Exp Ther Med. 2012;3(2):357–361. 22969896 10.3892/etm.2011.410 PMC3438633

